# Pediatric Venous Sinus Thrombosis Secondary to Cow’s Milk-Induced Iron-Deficiency Anemia

**DOI:** 10.7759/cureus.92394

**Published:** 2025-09-15

**Authors:** Sam I Hooshmand, Ahmad Sawalha, Marek Cierny

**Affiliations:** 1 Department of Neurology, Medical College of Wisconsin, Milwaukee, USA; 2 Department of Neurology, Bayhealth Medical Center, Milford, USA; 3 Department of Neurology, Philadelphia College of Osteopathic Medicine, Philadelphia, USA

**Keywords:** cow’s milk anemia, hypercoagulation, iron deficiency anemia, pediatric stroke, venous sinus thrombosis

## Abstract

Iron-deficiency anemia is a well-established but underrecognized risk factor for venous sinus thrombosis in both pediatric and adult populations. In young children, excessive cow’s milk consumption is a leading cause of dietary iron deficiency due to its low iron content and interference with iron absorption. We present a case of a 24-month-old male who developed extensive cerebral venous sinus thrombosis due to severe iron-deficiency anemia, arising from excessive cow’s milk intake and recent infection despite adequate complementary feeding. This case underscores the importance of early nutritional counseling, appropriate iron supplementation, and heightened clinical awareness of modifiable dietary risk factors in stroke prevention. Given the life-threatening nature of this complication and the frequency of excessive cow’s milk intake in toddlers, our report highlights a critical and preventable etiology of pediatric stroke.

## Introduction

Iron-deficiency anemia (IDA) is the most common nutritional deficiency, with a global prevalence of 16.7%, and is a significant cause of anemia in children, females, and residents of low socio-demographic index countries [1-3]. Despite its widespread prevalence, IDA is often underappreciated as a preventable risk factor for pediatric stroke [2-7]. Recent case-control studies and registry data demonstrate that over half of arterial ischemic strokes and cerebral venous sinus thromboses (CVST) in otherwise healthy children are attributable to IDA, with odds ratios for stroke ranging from 3.8 to 10 [2,4].

Nearly half of pediatric stroke cases involve CVST, which consistently shows an association with IDA in both children and adults [2-5,7]. The American Heart Association/American Stroke Association identifies IDA as an established risk factor for CVST in children, frequently interacting synergistically with other acquired prothrombotic conditions [2,4,7]. By detailing the clinical assessment and timely intervention in our case report, we aim to increase awareness of IDA as a preventable cause of pediatric CVST.

## Case presentation

**Figure 1:**
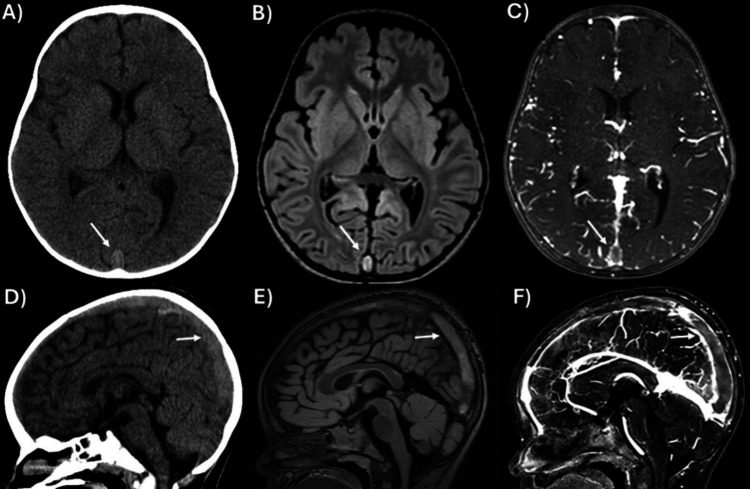
Cerebral Imaging at Presentation (A) Axial non-contrast CT shows a heterogeneous attenuated superior sagittal sinus (white arrow). (B) Axial FLAIR (Fluid Attenuated Inversion Recovery) shows thrombosis of the superior sagittal sinus (white arrow). (C) Axial magnetic resonance venography (MRV) shows thrombosis of the superior sagittal sinus (white arrows). (D) Sagittal non-contrast CT shows a heterogeneous attenuated superior sagittal sinus (white arrow). (E) Sagittal FLAIR shows thrombosis of the superior sagittal sinus (white arrow). (F) Sagittal MRV shows thrombosis of the cortical veins and the superior sagittal sinus.

A 24-month-old male presented to the emergency room with a seizure lasting one minute, characterized by right arm twitching and upward eye deviation. On arrival, the patient was at baseline but febrile (38.1°C). The family reported intermittent fevers for the past month, attributed to treated bilateral otitis media (OM), and denied previous seizures. Laboratory tests showed hemoglobin of 6.5 g/dL, mean corpuscular volume of 55.7 fL, and thrombocytosis of 612 k/µL. A non-contrast head CT revealed a heterogeneous superior sagittal sinus (Figure 1A, D). Brain MRI (magnetic resonance imaging)/MRV (magnetic resonance venography) confirmed thrombosis of the right transverse sinus, superior sagittal sinus, and adjacent cortical veins (Figure 1B, C, E, F).

**Figure 2:**
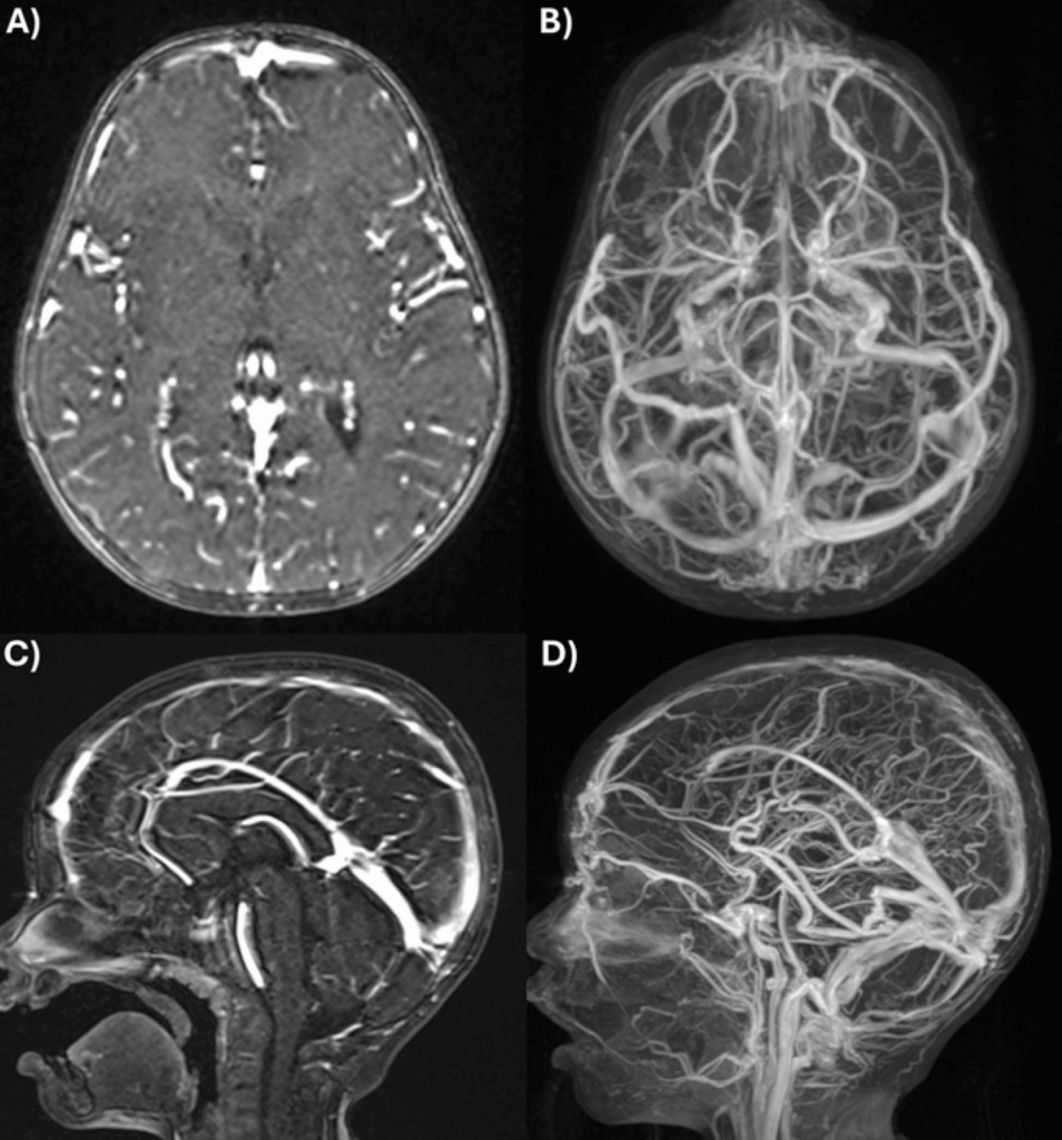
Cerebral Imaging at Three-Month Follow-up (A) Axial magnetic resonance venography (MRV) shows interval resolution of the filling defect. (B) Reformatted axial MRV shows near-complete resolution of thrombus in the superior sagittal sinus and right transverse sinus. (C) Sagittal MRV shows near-complete resolution of thrombus in the superior sagittal and sigmoid sinuses. (D) Reformatted sagittal MRV shows residual tubular partially occlusive thrombus in the anterior two-thirds of the sagittal sinus, although significantly decreased.

The patient was given a unit of packed red blood cells, started on anticoagulation with heparin, and supplemented with elemental iron. Lumbar puncture opening pressure was 36 mmH₂O, with unremarkable CSF analysis. Continuous video electroencephalogram was normal. History revealed daily cow’s milk (CM) consumption of approximately 24 oz (710 mL) and otherwise age-appropriate diet. Two days later, serum testing confirmed IDA. Parents were advised to discontinue CM and counseled on adequate complementary feeding. Imaging at three-month follow-up revealed near resolution of venous thromboses (Figure 2A-D). Low-molecular-weight heparin was discontinued five months after the initial presentation. 

## Discussion

The preventable risk factor of IDA receives less attention than traditional genetic or infectious etiologies of pediatric stroke, especially in high-income countries with a prevalence as low as 4% in North America [1]. This is despite its high burden, as up to half of all strokes in otherwise healthy children can be attributed to IDA [2,3]. Venous sinus thrombosis (VST) is implicated in almost half of pediatric stroke case series, with a trend toward association with IDA [2-4]. Case-control studies in adults have independently associated IDA with CVT [5,6]. IDA in the setting of recently treated OM was judged to be the cause of VST in our patient.

High CM intake causes IDA due to low iron content, occult intestinal blood loss, and inhibition of non-heme iron absorption by calcium and casein [8]. Current guidelines from the American Academy of Pediatrics do not suggest a specific limit for daily CM intake. However, some experts suggest that up to 24 oz (710 mL) in children older than twelve months is safe [9,10]. Our patient developed VST while consuming CM at the upper limit of this recommendation. Mechanisms underlying IDA and VST are not fully understood, but thrombocytosis and microcytosis in IDA reduce red cell deformability and may induce a hypercoagulable state [11].

## Conclusions

Our case illustrates a preventable, life-threatening consequence of IDA due to high CM intake of 24 oz (710 mL) per day. Early recognition of IDA and timely intervention through dietary counseling and iron supplementation are critical for primary prevention. For children older than twelve months, we propose CM intake of no more than 12 oz (355 mL) to allow a safe margin to avoid the life-threatening complications observed in our case report. We recommend that pediatric societies consider establishing a specific, evidence-based upper limit for CM intake in children older than twelve months, with an appropriate margin of safety to mitigate the risk of serious complications, including stroke.
